# Complete solid-body rotation rate measurements of micro-plastic curved fibers in turbulence

**DOI:** 10.1007/s00348-025-04021-0

**Published:** 2025-05-02

**Authors:** Giuseppe C. A. Caridi, Vlad Giurgiu, Marco De Paoli, Alfredo Soldati

**Affiliations:** 1 Institute of Fluid Mechanics and Heat Transfer, TU Wien, 1060 Vienna, Austria; 2 Physics of Fluids Group, University of Twente, 7500AE Enschede, The Netherlands; 3 Polytechnic Department, University of Udine, 33100 Udine, Italy

## Abstract

**Abstract:**

In this study we quantify the uncertainty relative to a novel Lagrangian tracking technique to measure the complete solid-body rotation rate of anisotropic micro-plastic fibers. By exploiting their geometry—specifically, their elongation and curvature for tumbling and spinning rate measurements, respectively—we address a gap in the literature regarding the tracking of fibers’ unique orientation along their trajectories. The impact of fiber geometry and imaging parameters on the accuracy of the solid-body rotation rates measurements is investigated. The influence of spatial and temporal resolution on the measurement uncertainty is assessed on synthetic data. Experimental results obtained in a channel flow demonstrate the method’s potential to accurately detect rotations of fibers with lengths approaching the Kolmogorov scale.

**Graphical abstract:**

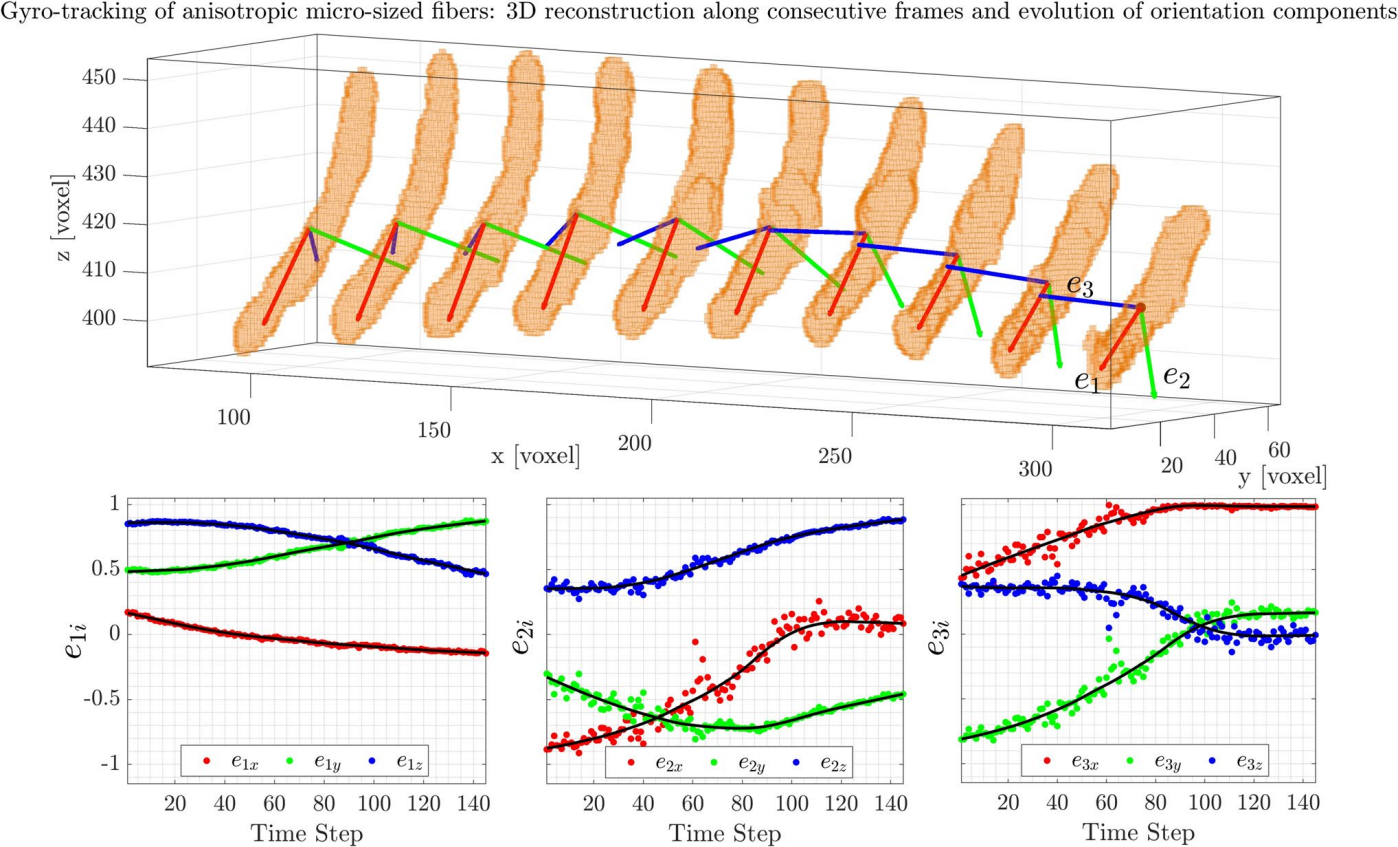

## Introduction

The dynamics of small and anisotropic particles in turbulent flows is a growing field of study. Understanding the behavior of anisotropic particles in turbulent flows is crucial due to its numerous industrial applications, such as in pulp and papermaking (Lundell et al. 2011) and catalysis with nanofibers (De Jong and Geus 2000). In addition, environmental processes addressing micro-plastic pollution are currently the focus of significant global concern and extensive research studies (do Sul and Costa 2014; Horton et al. 2017; Rillig et al. 2017; Ross et al. 2021). Among all different types of anisotropic particles, fibers have significant importance due to their widespread presence. Specifically, synthetic fibers appear to be ten times more abundant than other types of micro-plastics in the environment and represent the predominant shape ingested by marine biota (Ugwu et al. 2021). Worth to mention, washing a single piece of synthetic clothing can release over 1900 strands (Ziani et al. 2023).

Over the past decades, numerical modeling of micro-plastic transport, particularly for anisotropic particles such as fibers, in oceans and rivers has advanced significantly. However, critical parameters in these models, such as settling velocity, drag, and particle-turbulence interactions, remain partially unexplored. Consequently, accurately modeling the settling and dispersion of micro-plastics in the environment remains challenging and is beyond current capabilities (Voth and Soldati 2017; Brandt and Coletti 2022). To be specific, as the degree of particle anisotropy increases, so too does the number of unanswered fundamental questions. The shape of the particles exerts a significant influence on their orientation and rotation, which in turn affects the interactions between particles and the surrounding fluid. The addition of the shape in the particle parameters list (size, density, concentration) has marked a turning point in numerical simulations and definition of boundary conditions (Prosperetti 2015).

**Fig. 1:**
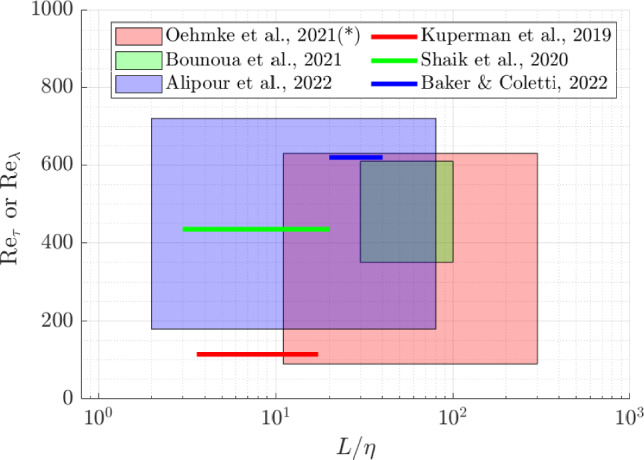
A survey of the parameters space explored in previous studies in terms of fiber length normalized by the Kolmogorov length scale $(L/\eta )$ and of the Reynolds number. In wall-bounded flows, the shear Reynolds number (Re$_\tau$) is indicated, while in homogeneous and isotropic turbulence, the Taylor Reynolds number (Re$_\lambda$) is considered ($^*$)

For these reasons, with the growing demand for empirical data and the advancement of optical measurement techniques, experimental research on fiber-turbulence interaction has recently been on the rise. The current challenge lies in measuring not only the particle velocity in turbulent flows but also its orientation and rotational rate in order to obtain the complete dynamics and eventually the response of a fiber to changes in the surrounding flow. These challenges are primarily addressed through experimental studies conducted in homogeneous isotropic turbulence (HIT) or wall-bounded flow environments. Preliminary studies utilize 2D PIV measurements to obtain the mean and fluctuating velocities of both phases (fibers and carrier fluid), as well as the angular velocities of the fibers within the laboratory reference system (Hoseini et al. 2015; Capone et al. 2017, 2021; Giurgiu et al. 2024a). On the other hand, three-dimensional reconstruction methods are needed to obtain the intrinsic behavior of fibers within their reference system (e.g., spinning and tumbling rotational rates). Baker and Coletti (2022a) used a hybrid technique to obtain the three-dimensional orientation of fibers from 2D measurements. However, recent studies have only allowed to measure the tumbling rates, due to the axial symmetry of the cylindrical fibers employed. Shaik and van Hout (2023) used a two-orthogonal view digital holography system to detect all three components of $\textbf{e}_1$, i.e. the vector aligned with the longitudinal fiber axis, in a turbulent channel flow. The orientations of the fiber projections onto the two cameras were used for this purpose, and tumbling rates were computed similarly to Baker and Coletti (2022b). Both investigations are limited to measurements of tumbling rates only. To measure the spinning rate, it is necessary to break this symmetry and employ a three-dimensional reconstruction approach. This was first achieved by placing markers on the fiber’s surface. Oehmke et al. (2021) measured both spinning and tumbling rates of fibers in HIT using three cameras. These cameras captured images of fibers with a printed helix, allowing the reconstruction of fiber’s position and the orientation of its major axis. Will et al. (2021) used a similar approach by painting buoyant spheroids with a black and white pattern to identify their orientation while rising in a still fluid. The use of markers and surface patterns requires large particles (typically $\mathcal {O}(1)$ cm in size), which can limit their applicability in various contexts. This limitation is particularly significant when the particle dimensions need to be on the order of, or smaller than, the Kolmogorov length scale. Notably, even when particles are in the tracing regime (characterized by neutral buoyancy and small size, see Brandt and Coletti 2022) they exhibit complex orientational dynamics while following Lagrangian trajectories. This complexity stems from the coupling between their rotation and the velocity gradients within the turbulent field, presenting a compelling application of the fundamental properties of velocity gradients in turbulence. In contrast, inertial particles exhibit preferential concentration and alignment (Voth and Soldati 2017). Both regimes, however, require further grounding in the fundamental physics governing non-spherical particles. To advance research in this area, it is crucial to focus on tracer particles as well. To this end, breaking the symmetry of particles through their geometry is advantageous, as it enables the unambiguous detection of their orientation during imaging. Marcus et al. (2014) demonstrated this by measuring the orientation and rotation rates of two types of 3D-printed anisotropic particles, namely crosses and jacks, respectively corresponding to two and three rods mutually perpendicular and connected at their mid points. Similarly, Alipour et al. (2021, 2022) and Giurgiu et al. (2024b) explored the rotational dynamics of curved slender fibers. A summary of the parameters space covered in these works and expressed in terms of fibers length relative to the Kolmogorov length scale and Reynolds number is reported in Fig. 1.

As a result of the growing attention to the characterization of the rotation rates of anisotropic particles in turbulence, 3D imaging tracking techniques are increasingly advancing to encompass rotational measurements, alongside traditional translational metrics such as velocity and acceleration. This evolution necessitates a thorough definition of measurement methodologies, particularly concerning the repeatability and the optimization of rotational accuracy, a progression akin to the advancements previously achieved in particle image velocimetry (PIV) and particle tracking velocimetry (PTV) (Raffel et al. 2018; Sciacchitano 2019; Charonko and Vlachos 2013). These measurements typically require a complex array of parameters to be determined during the experimental design phase. For PIV and PTV, the key factors include spatial and temporal resolution, where the former is influenced by magnification and pixel resolution of the camera. The latter depends on the acquisition frequency in single-frame mode, which in turn defines the angular shift for a given particle’s rotational rate. The precision in measuring a particle’s position and displacement is intrinsically tied to these parameters, as previously established by Raffel et al. (2018), and the same applies to the orientation and angular displacement of particles.

Measurements of spinning rates of curved fibers have been previously presented (Giurgiu et al. 2024b). In present work, we provide a detailed description of the procedure employed, highlighting and analyzing also the primary sources of error. Furthermore, by applying this measurement technique to synthetic data, we demonstrate how the uncertainty of such measurements varies with respect to specific parameters such as fiber length, curvature, spatial resolution, and angular displacement. Additionally, through the visualization and analysis of individual trajectories, we conclude that the principal source of error in real experiments arises from 3D reconstruction noise, which directly affects the orientation components of the rotational matrix, rather than the mathematical approach (rotational matrix or quaternion). We consider temporal filtering techniques used in previous studies to mitigate these errors, and we also propose an alternative solution based on the Iterative Closest Point (ICP) algorithm.

## Measurement of full body rotation using 3D imaging techniques

Measuring the rotation of a rigid body using a 3D imaging system involves several critical steps. These include setting up the time-resolved imaging system, capturing the motion of the object, and processing the captured images with a 3D reconstruction algorithm. The following step consists of the phase discrimination, where objects of interest are identified and distinguished from tracers and other spurious reconstructed entities. At this stage, it is possible to define the rigid body’s properties, such as shape, volume, center of mass, and orientation. Parameters like volume and aspect ratio can be particularly useful during the phase discrimination process.

Subsequently, the trajectory of the object is determined by linking the 3D positions of the center of mass across consecutive frames to form continuous trajectories. This requires solving the correspondence problem, ensuring that the same object is accurately identified at each successive time step. Various algorithms, including nearest neighbor, predictive tracking, or global optimization, can be employed for this purpose. Once the trajectory is established, the velocity is determined from the displacement of the center of mass, and the rotational rate is calculated from the angular displacement of the object’s orientation.

Regarding measurements of translational velocity, an extensive body of literature spanning over 30 years already exists. During this time, from early research (Nishino et al. 1989; Maas et al. 1993) to the most recent advancements (Schanz et al. 2016; Schröder and Schanz 2023), much discussion has centered around optimizing measurement settings and parameters to improve accuracy and precision. The readers are referred to these works, as well as fundamental studies on the sources of errors related to image particle displacement (Raffel et al. 2018). In the following discussion, the effect of the number of trajectories or particle concentration (i.e., particle per pixel, PPP) will not be considered; instead, we will focus solely on the accuracy of rotation rate measurement along individual trajectories. The procedure to retrieve orientation and angular displacement, along with the associated sources of error, is detailed below, with particular emphasis on particles exhibiting a fiber-like shape. While the following discussion may at times appear elementary or familiar, it serves to identify the sources of error, which will be examined in Sect. 2.2.

### Determination of the rotation rates

**Fig. 2:**
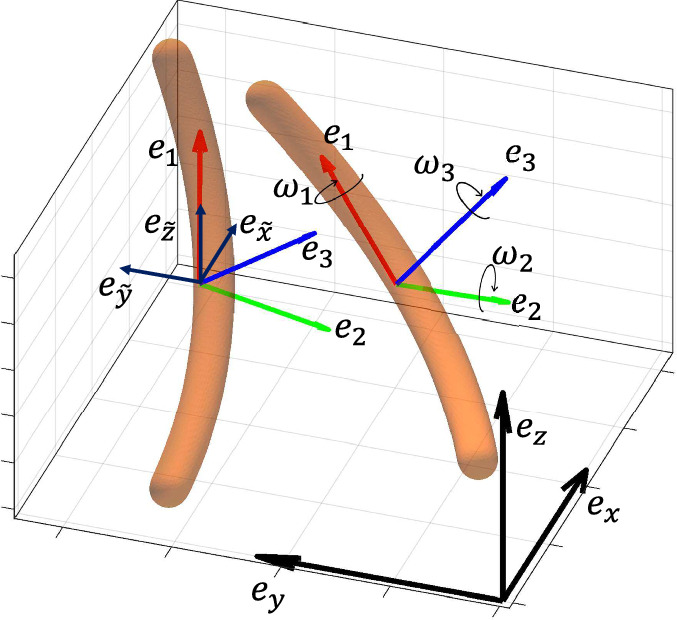
Sketch of a synthetic slender fiber undergoing both rotation and translation. Laboratory reference frame is represented by ($\textbf{e}_{\textbf{x}}$, $\textbf{e}_{\textbf{y}}$, $\textbf{e}_{\textbf{z}}$), laboratory reference frame translated to the fiber’s centroid by ($\textbf{e}_{\tilde{\textbf{x}}}$, $\textbf{e}_{\tilde{\textbf{y}}}$, $\textbf{e}_{\tilde{\textbf{z}}}$), while the fiber-fixed axes are denoted by ($\textbf{e}_{\textbf{1}}$, $\textbf{e}_{\textbf{2}}$, $\textbf{e}_{\textbf{3}}$)

Consider the 3D reconstruction of a fiber, defined by a set of *n* points (or voxels), each having coordinates in the laboratory reference frame $\textbf{p}_j = (x_j, y_j, z_j)$ with $1\le j\le n$, which is transported within a generic flow field, as illustrated in Fig. 2. The fiber’s reference frame is centered at its centroid, with coordinates $\textbf{r}_c = (x_c, y_c, z_c) = n^{-1} \sum _{j=1}^n (x_j, y_j, z_j)$. In the laboratory reference frame, translated to the fiber’s centroid, the set of *n* points is represented by coordinates $\tilde{\textbf{p}}_j = (\tilde{x}_j, \tilde{y}_j, \tilde{z}_j) = (x_j - x_c, y_j - y_c, z_j - z_c)$. The inertial coordinates, corresponding to the laboratory reference frame, are aligned with the unit vectors ($\textbf{e}_{\textbf{x}}$, $\textbf{e}_{\textbf{y}}$, $\textbf{e}_{\textbf{z}}$), while ($\textbf{e}_{\textbf{1}}$, $\textbf{e}_{\textbf{2}}$, $\textbf{e}_{\textbf{3}}$) represent the principal directions of inertia of the fiber in its own reference frame. These principal directions are determined via eigenvalue decomposition of the inertia tensor $\textbf{I}$ computed with respect to the fiber’s centroid, and are expressed in the laboratory reference frame translated to the centroid:

1 $$\begin{aligned} \textbf{I}=\sum _{j=1}^{n} m_j \begin{bmatrix} (\tilde{y}^2_j + \tilde{z}^2_j) & -\tilde{x}_j \tilde{y}_j & -\tilde{x}_j \tilde{z}_j \\ -\tilde{x}_j \tilde{y}_j & (\tilde{x}_j^2 + \tilde{z}_j^2) & -\tilde{y}_j \tilde{z}_j \\ -\tilde{x}_j \tilde{z}_j & -\tilde{y}_j \tilde{z}_j & (\tilde{x}_j^2 + \tilde{y}_j^2) \\ \end{bmatrix} \end{aligned}$$

where $m_j$ is set to unity. The eigenvalue decomposition of $\textbf{I}$ is given by:

2 $$\begin{aligned} \textbf{I} \textbf{e}_i= \lambda _i \textbf{e}_i \end{aligned}$$

where $\lambda _i$ are the eigenvalues and $\textbf{e}_i$ are the corresponding eigenvectors, representing the principal directions of inertia.

To represent the current fiber orientation, we build the rotation matrix $\textbf{R}$, which relies on the principal directions of inertia of the fiber (Lynch and Park 2017):

3 $$\begin{aligned} \textbf{R}= \begin{bmatrix}\textbf{e}_1&\textbf{e}_2&\textbf{e}_3\end{bmatrix}= \begin{bmatrix} e_{1x} & e_{2x} & e_{3x} \\ e_{1y} & e_{2y} & e_{3y} \\ e_{1z} & e_{2z} & e_{3z} \\ \end{bmatrix} \text { .} \end{aligned}$$

This matrix can also be considered an orientation matrix, analogous to the position vector in linear motion. However, to maintain consistency with established nomenclature in the field, we will continue to refer to it as the “rotation matrix”. In this formulation, although nine components appear, only three result being independent due to the constraints of orthogonality and normalization, which ensure length preservation. Consequently, the rotation rate of a particle along its trajectory is determined by the change in its orientation over time:

4 $$\begin{aligned} \dot{\textbf{R}} = \frac{d\textbf{R}}{dt} \approx \frac{\textbf{R}(t+\Delta t) - \textbf{R}(t-\Delta t) }{2\Delta t}\text { ,} \end{aligned}$$

where *t* is the current time and $\Delta t$ is the time separation between two consecutive instants available.

To express the angular velocities from the rotation rate matrix $\dot{\textbf{R}}$, the skew-symmetric matrix ${\varvec{\Omega }}_{xyz}$ is used, according to the following relation (Goldstein 2011):

5 $$\begin{aligned} {\varvec{\Omega }}_{xyz}= \begin{bmatrix} 0 & -\omega _{z} & \omega _{y} \\ \omega _{z} & 0 & -\omega _{x} \\ -\omega _{y} & \omega _{x} & 0 \\ \end{bmatrix}=\dot{\textbf{R}}\textbf{R}^{-1}=\dot{\textbf{R}}\textbf{R}^{T}\text { .} \end{aligned}$$

where $\omega _x$, $\omega _y$, and $\omega _z$ represent the rotation rates of the fiber with respect to the laboratory reference frame. Similarly, the matrix ${\varvec{\Omega }}_{123}$, which contains the rotation rates around the axes of the fiber reference frame, is defined as:

6 $$\begin{aligned} {\varvec{\Omega }}_{123}=\begin{bmatrix} 0 & -\omega _{3} & \omega _{2} \\ \omega _{3} & 0 & -\omega _{1} \\ -\omega _{2} & \omega _{1} & 0 \\ \end{bmatrix}=\textbf{R}^{T}\dot{\textbf{R}}\text { .} \end{aligned}$$

Here, the tumbling motion of the fiber is defined as $\omega _t = \sqrt{\omega _{2}^2 + \omega _{3}^2}$, while the spinning motion is given by $\omega _s = \omega _{1}$. Note that the total rotation rate is an invariant, i.e. it remains the same when computer in the two reference frames considered, i.e. $\omega _t^2+\omega _s^2=\omega _{1}^2 + \omega _{2}^2 + \omega _{3}^2 = \omega _{x}^2 + \omega _{y}^2 + \omega _{z}^2$ (Voth and Soldati 2017).

An additional method to compute the rotations consists of the quaternions (Evans 1977; Eberly 2002; Zhao and Van Wachem 2013), a mathematical representation of orientation using four parameters and grouped within the symbol $\textbf{q}$, to define the 3D orientation of an object as:

7 $$\begin{aligned} \textbf{q} = w + q_1 \textbf{i} + q_2 \textbf{j} + q_3 \textbf{k}\text { ,} \end{aligned}$$

where *w* is the scalar component that relates to the angle of rotation (i.e., cosine of half the rotation angle) and $(q_1,q_2,q_3)$ represent the vector components, corresponding to the axis of rotation. The unit vectors $\textbf{i},\textbf{j},\textbf{k}$ are basis elements with special multiplication rules $\textbf{i}^2=\textbf{j}^2=\textbf{k}^2=\textbf{i}\textbf{j}\textbf{k}=-1$ (they represent the directions in 3D space, analogous to the *i* in complex numbers, but extended to three dimensions). With this mathematical representation, the orientations of a fiber in two consecutive instants can be expressed by two quaternions: $\textbf{q}_{\textbf{t}}$ and $\textbf{q}_{\textbf{t} + {\varvec{\Delta }} \textbf{t}}$. The scalar and vector components at every instant need also to be computed from the Euler angles or principal axis components. Therefore the relative quaternion $\textbf{q}_{\textbf{r}}$ which represents the rotation from the two consecutive orientations is:

8 $$\begin{aligned} \textbf{q}_{\textbf{r}} = \textbf{q}_{\textbf{t} + {\varvec{\Delta }} {\textbf{t}}} \times \textbf{q}_{\textbf{t}}^{-1} \end{aligned}$$

where $\textbf{q}_{\textbf{t}}^{-1}= (w, -q_1, -q_2, -q_3 )$ at time *t*. The rotation angle $\theta _r$ is computed from the scalar part $w_r$ of $\textbf{q}_{\textbf{r}}$, while the axis of rotation $\textbf{u}_{\textbf{r}}$ from the vectorial part, as:

9 $$\begin{aligned} \theta _r = 2 \cos ^{-1}(w_r)\quad ,\quad \textbf{u}_{\textbf{r}}= \frac{(q_{r,1},q_{r,2},q_{r,3})}{\sin (\theta /2)} \end{aligned}$$

Finally, the angular velocity $\varvec{\omega }$ is obtained as:

10 $$\begin{aligned} \varvec{\omega }= \frac{\theta _r}{\Delta t}\textbf{u}_{\textbf{r}}\text { .} \end{aligned}$$

It should be noticed that Eq. (8) is not affected by singularities, making it more robust compared to Eq. (4). This is why this method is more commonly used. The angular velocities in the reference frame of the fiber are obtained using the rotational matrix $\textbf{R}$:

11 $$\begin{aligned} (\omega _1,\omega _2,\omega _3)= \textbf{R}^T \varvec{\omega }\text { .} \end{aligned}$$

### Main source of errors in full body rotation measurements

When measuring angular velocities, experimental errors can be categorized into two primary sources: those arising from the determination of the object’s orientation, and those related to the computation of the rotational rate, which we will describe in Sect. 2.2.1 and Sect. 2.2.2, respectively.

#### Errors in orientation calculation

**Fig. 3:**
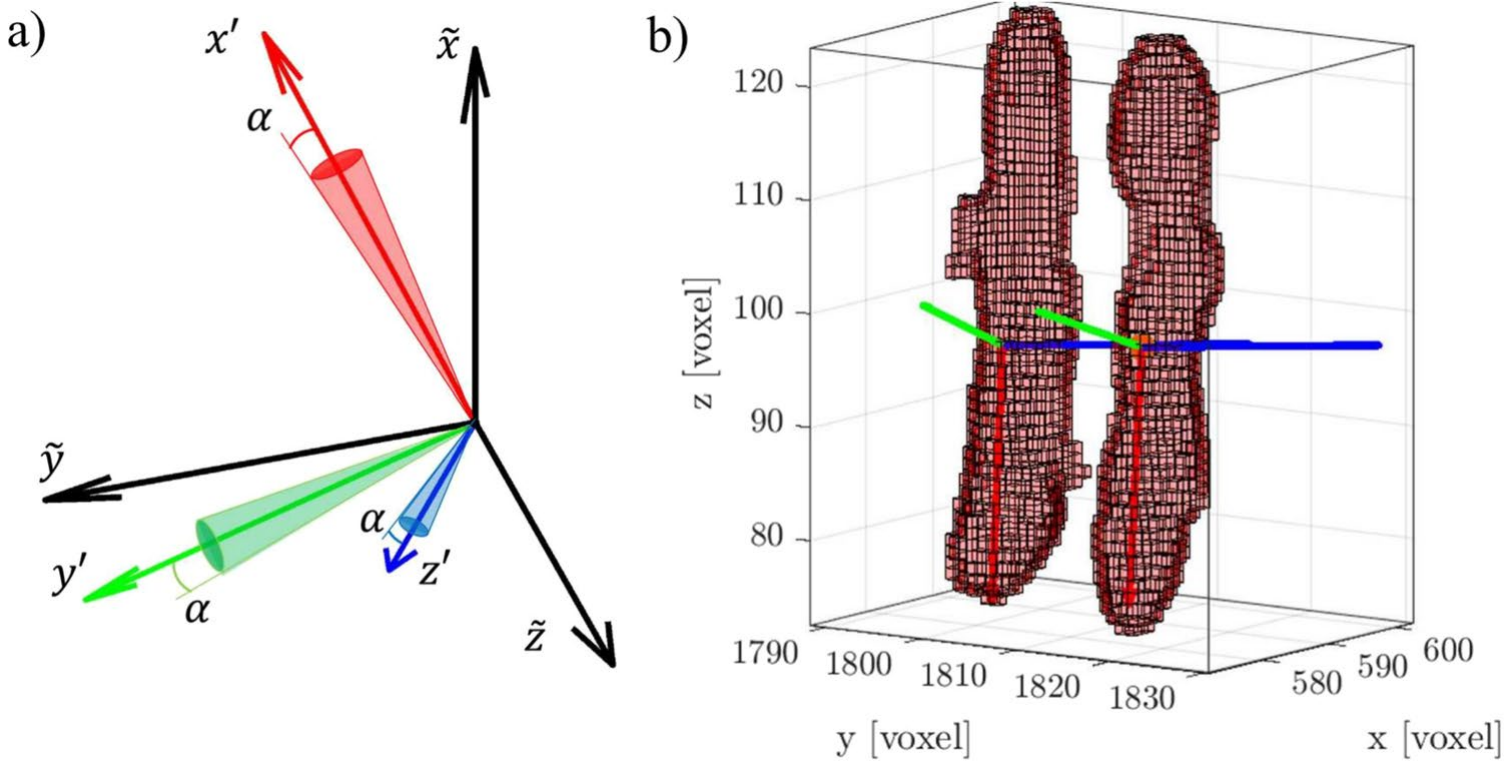
**a** General representation of the orientation error $\alpha$ on the fiber’s reference system ($x^\prime y^\prime z^\prime$). **b** Illustration of interface noise obtained in a 3D reconstruction of a real fiber from the experiments by Giurgiu et al. (2024b)

All the methods that could be used to compute the rotation rate rely on the fiber’s instantaneous orientation (i.e., $\textbf{R}$ or $\textbf{q}$). It is essential to assess the experimental errors affecting this step, similar to those encountered when defining the center of mass in PIV or PTV calculations. These errors, denoted here by $\alpha$, impact the instantaneous orientation measurement of the particle, as shown in Fig. 3a. As Eqs. (1) and (2) demonstrate, the principal axes of the fiber depend on the distribution of the point cloud $\textbf{p}_j$, particularly the discretization in voxel (resolution) and the 3D reconstruction noise. For example, if the resolution is insufficient to fully capture the object’s shape (especially for anisotropic geometries), the point cloud $\textbf{p}_j$, representing the spatial coordinates of the center of mass of each voxel occupied by the object, may lead to distorted properties, such as an inaccurate center of mass or misaligned principal axes orientation.

Additionally, experimental noise introduces imperfections at the particle interfaces, as seen in Fig. 3b. The source of this noise may be attributed to light-particle interactions, background noise in the raw images, as well as the reconstruction algorithm used. Figure 3b shows an example of interface noise which appears and disappears across two consecutive frames of the same fiber. Such noise is discontinuous in time and alters the imaged mass distribution (i.e., shape). Consequently, both the inertia tensor and eigenvalue decomposition are affected by resolution and interface noise, leading to errors in the principal axes ($\textbf{e}_{\textbf{1}}$, $\textbf{e}_{\textbf{2}}$, $\textbf{e}_{\textbf{3}}$) which are used to define the orientation with the rotation matrix $\textbf{R}$ with Eq. (3), as well as to compute the orientation in quaternion units.

Finally, for slender objects like fibers, an ambiguity arises in defining the two eigenvectors $\textbf{e}_{\textbf{2}}$ and $\textbf{e}_{\textbf{3}}$ as the curvature approaches zero (indicating straight fibers). As a result, depending on the fiber’s resolution, there exists a minimum curvature value $(K^*_{min})$ below which the orientation of these eigenvectors becomes uncertain. Similarly, there is a critical maximum curvature value $(K^*_{max})$, where the height and width of the fiber arc become nearly identical (i.e., the fiber resembles: a circular shape). In this scenario, the eigenvectors $\textbf{e}_1$ and $\textbf{e}_2$ can interchange, leading again to ambiguity in the orientation. To assess and quantify this ambiguity, the values of the three eigenvalues (denoted as $\lambda _i$ in Eq. (2)) can be compared with one another. When an ambiguity exists between two principal axes, their corresponding eigenvalues tend to be very similar. The minimum and maximum curvature values at which the ambiguity of the principal axis is avoided are presented in Sect. 3.

#### Errors in rotation rate calculation

For both rotational matrix and quaternion method, one of the most important sources of error is related to the time discretization. In the finite difference approximation of the time derivative of the rotation matrix (Eq. (4)), discretization errors are known to increase with $\Delta t$ due to truncation error. Although smaller time steps improve the accuracy of the approximation, they also amplify sensitivity to numerical errors, such as round-off errors. Moreover, when the angular displacement is of the same order of magnitude as the orientation error (i.e., $\alpha$), its measurement results in a noisy output. Similarly, when calculating the angular velocity using quaternions, the relative quaternion between two time steps is used. For large $\Delta t$, the assumption that angular velocity remains constant during the interval introduces inaccuracies, and for small rotation angles the calculated rotation rate becomes increasingly susceptible to orientation errors.

Another source of error is the presence of singularities, such as the Gimbal lock, leading to undefined or ambiguous results (Hemingway and O’Reilly 2018). While rotation matrices are inherently free from singularities like Gimbal lock, extracting Euler angles from a rotation matrix can introduce singularities, particularly when the rotation involves critical angles (e.g., near 90$^{\circ }$ for certain Euler angles). Conversely, quaternions do not suffer from Gimbal lock due to their four-dimensional structure, which allows for the smooth representation of all possible rotations without any loss of degrees of freedom. Consequently, quaternions are generally favored for avoiding singularities and providing a continuous, robust representation of rotations in 3D space (Evans and Murad 1977).

## Numerical assessment

A number of parameters, including 3D reconstruction noise, curvature, angular displacement and resolution, affect the rotational rate uncertainty. In this section, we present the a priori estimation of uncertainty in the measured rotation, rather than the rotational rate, as our techniques directly provide the former. Distinguishing between these quantities is essential, akin to the relationship between particle displacement and velocity in PIV measurements, where the rate is obtained by dividing the measured rotation by the constant time interval between consecutive frames. The data are generated by varying one parameter at a time, such that the procedure ensures a full control of the parameters considered allowing to infer their effect on the rotational error. We chose to use synthetic particles as it is a well-established approach to quantify the PIV uncertainty (Kähler et al. 2012; Xue et al. 2015; Sciacchitano 2019).

**Fig. 4:**
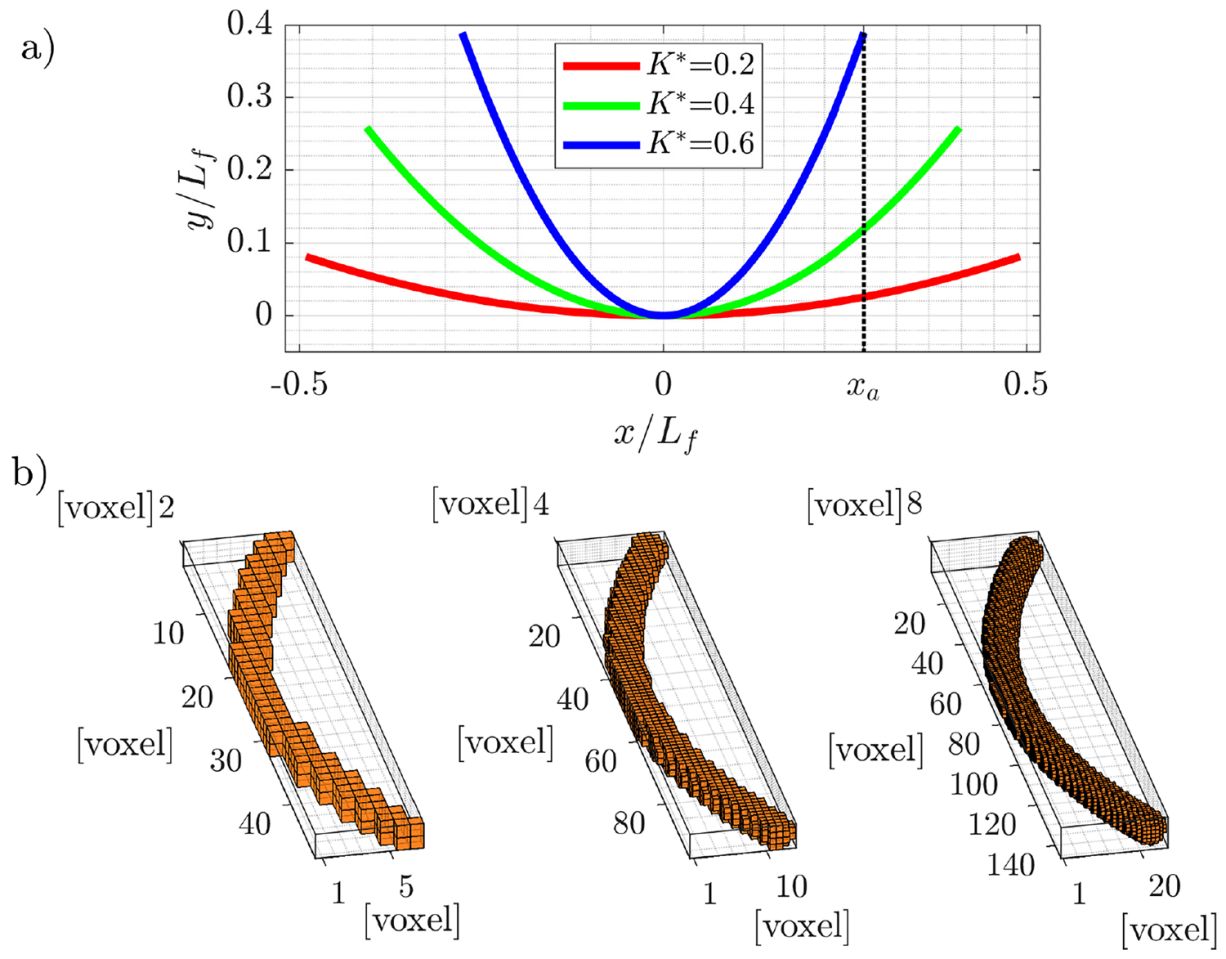
**a** Three examples of polynomials used to generate synthetic fibers with dimensionless curvatures $K^*$ of 0.2, 0.5, and 0.6. **b** 3D reconstruction of the synthetic fiber with $K^*=0.3$ at three different resolutions, increasing from left to right: 50, 100, and 150 voxels per unit length

Synthetic fibers are generated using a second-order polynomial function to define their axis, implying that the fibers’ axis is contained on a plane (Alipour et al. 2021). In a *x*, *y* reference frame, the polynomial is assumed to be symmetric about the apex located at $x=0$ and is represented in the simplified form $y=ax^2$. The endpoints (located at $x=\pm x_a$) and the coefficient *a* are determined using the definitions of mean curvature and arc length of the parabola. The dimensionless curvature $K^*=K/K_0$, used to characterize the fiber, is defined as in Alipour et al. (2021), where the mean curvature *K* is normalized by $K_0$, which is determined by considering the curvature of a semicircular arc with a length equal to the length of the fibers, $L_f$. Specifically, $K_0=\pi /L_f$. The polynomials for the fibers were generated to have values of $K^*$ ranging from 0.05 to 0.9. Three examples of fibers’ shape polynomial are shown in Fig. 4a. To generate the full 3D voxel distribution, a sphere with a given diameter $D_f$ is placed at each point along the polynomial curve, creating a continuous 3D structure. Here $D_f$ is chosen such that the resulting aspect ratio is $L_f/D_f=20$. The fibers are then discretized at three different resolutions: 50, 100, and 150 voxels along the fiber’s axis (see the illustration in Fig. 4b).

**Fig. 5:**
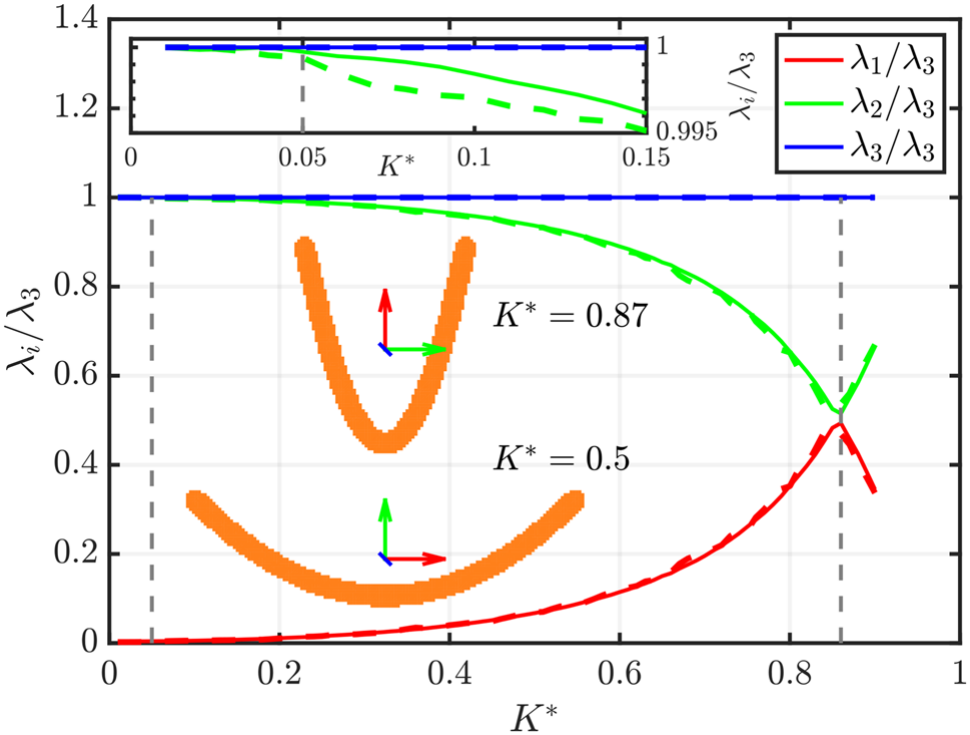
Comparison of $\lambda _1$, $\lambda _2$, and $\lambda _3$ as a function of curvature $K^*$. Values are normalized by $\lambda _3$ for visualization purposes. Solid lines represent the values obtained for fibers at a resolution of 150 voxels, while dashed lines correspond to a resolution of 50 voxels. Dashed vertical line indicates the curvature values at which the principal axes switch, marking the ambiguity conditions ($K^*=0.05$ and 0.83). A close-up view of the low-curvature region is reported in the inset. Two exemplar fibers, corresponding to $K^*=0.5$ and $K^*=0.87$ are also reported

Before computing the uncertainty related to the rotational measurement, it is necessary to find the values of $K^*_{min}$ and $K^*_{max}$, defined in Sect. 2.2.1, to establish the range of curvature in which the orientation computation is unaffected by the interchange of principal axes. As an illustration, Fig. 5 shows $\lambda _1$, $\lambda _2$, and $\lambda _3$ as a function of curvature $K^*$. The values of $\lambda _2$ and $\lambda _3$ become similar at $K^*\approx 0.05$, while for $\lambda _1$ and $\lambda _2$ the critical curvature is reached at $K^*\approx 0.83$. These limits appear to be independent of resolution, as the solid and dashed lines (representing resolutions of 150 and 50 voxels, respectively) are nearly overlapping. In the following discussion, only the curvature range $0.05<K^*<0.6$ is considered. Fibers in the range $0.6<K^*<0.83$ are excluded, as they deviate from the slender shape. As discussed by Giurgiu et al. (2024b), fibers with $K^*<0.6$ exhibit a behavior similar to that of straight fibers.

**Fig. 6:**
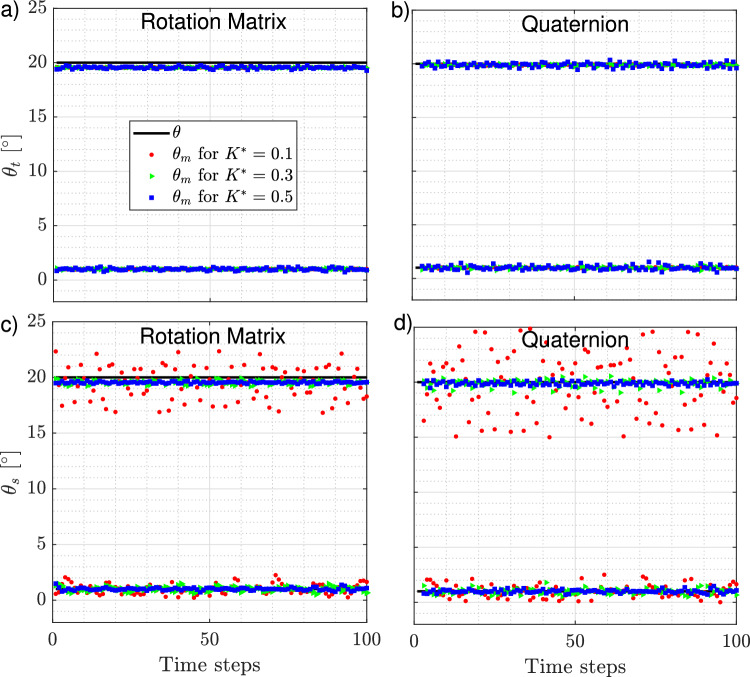
Comparison of tumbling ($\theta _t$) and spinning ($\theta _s$) angular displacements with the measured displacement ($\theta _m$), computed via rotation matrix (**a**, **c**) and quaternions (**b**, **d**). The values considered are $\theta = 1^\circ$ and $20^\circ$ for the angular displacement, $K^* = 0.1$, 0.3, and 0.5 for curvature, and a resolution of 100 voxels

To assess the uncertainty estimation, a constant angular displacement $\theta$ is applied with values ranging from 0.05 to 45 degrees per time step, over $N=100$ time steps. Specifically, $\theta _s$ is applied around the principal axis $\textbf{e}_1$ and $\theta _t$ around $\textbf{e}_2$ to assess errors associated with measurements of the spinning and tumbling rates, respectively. For each trajectory, the rotation in the fiber’s reference frame is computed to obtain measurements of the angular displacement $\theta _m$ using the rotation matrix and quaternion methods (Eq. (6) and Eq. (10)). A second-order central scheme was used to compute the finite difference in Eq. (4). Examples of the resulting $\theta _m$ are shown in Fig. 6 for synthetic fibers with curvatures of $K^* = 0.1$, 0.3, and 0.5, and a resolution of 100 voxels. In each panel, two different rotation rates are considered: a value corresponding to $1^\circ$/time step, and a larger value of $20^\circ$/time step. Panels *a* and *c* display the measurements obtained using the rotation matrix, while panels *b* and *d* show those obtained with quaternions. These results reveal that the disparity between the applied rotation and the measured rotation, $\theta _{t,s} - \theta _m$, can manifest as both mean disparity (or offset) and fluctuating component. The offset is particularly noticeable at larger angles, especially when using the rotation matrix to measure $\theta _m$ (Fig. 6a, c). Additionally, this offset appears independently of the fiber’s curvature. The fluctuating component of the disparity is more pronounced in the spinning component of the rotation and increases for fibers with low curvature ($K^* = 0.1$), particularly when the quaternion method is used compared to the rotation matrix (Fig. 6c, d).

For these reasons, the error introduced by the discretization of voxels, curvature, and angular displacement, is analyzed separately for mean and fluctuating component. The latter scales with the standard deviation $\sigma$ of the measured angular displacement $\theta _m$, and is inversely proportional to the square root of the number of samples, $N = 100$, assuming a Gaussian distribution of the angular displacement along each trajectory. Thus, the random error can be estimated as $\sigma /\sqrt{N}$ (Coleman and Steele 2018). As it was shown, the mean of the disparity (systematic error) $\mu$ may be nonzero (when detectable bias errors are present), and therefore it should be accounted.

**Fig. 7:**
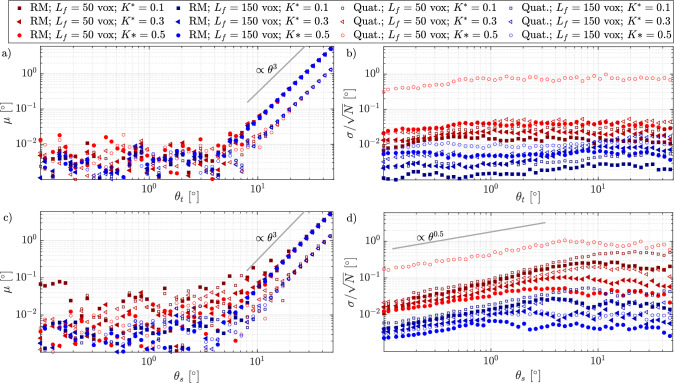
The absolute systematic, $\mu$, and random, $\sigma /\sqrt{N},$ errors as a function of the applied rotation for both tumbling (panels **a** and **b**) and spinning (panels **c** and **d**)

Figure 7 presents the systematic and random errors as functions of the applied rotation for both tumbling (panels **a** and **b**) and spinning (panels **c** and **d**). The data correspond to fibers with low (red markers) and high resolution (blue markers), measured across three different curvatures. The rotation is computed using both the Rotational Matrix (RM) and quaternion (Quat.) methods. The systematic error exhibits two distinct behaviors that depend solely on the applied angle. Specifically, for $\theta _t < 10^\circ$, the error oscillates around values smaller than $10^{-2}$. However, for $\theta _t > 10^\circ$, the error increases with $\theta ^3$. Importantly, neither the resolution nor the curvature significantly influence the systematic error, especially for larger angles, where all types of fibers follow the same power-3 law. The choice of rotation computation method, RM or quaternion, affects the systematic error only at larger angles, with the quaternion method yielding an average error approximately five times smaller (see Fig. 7a). In contrast, for spinning rotation, the effect of resolution on $\mu$ is evident at smaller angles, where the data from fibers with 50 voxels/$L_f$ (red markers) appear above those with 150 voxels/$L_f$ (blue markers). Furthermore, low-resolution fibers follow a power-3 law at larger angles, approximately at $\theta _s = 15^\circ$, while fibers with 150 voxels/$L_f$ maintain low values of $\mu$ up to $\theta _s = 8^\circ$ before aligning with the power-3 law, as shown in Fig. 7c.

**Fig. 8:**
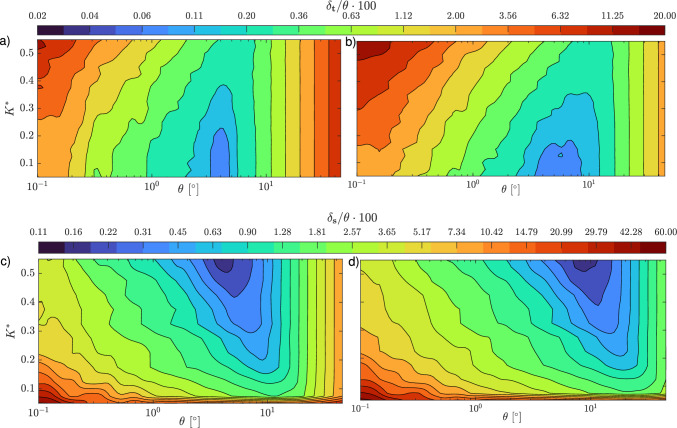
Contours of the relative total error $\delta$ defined in Eq. (12) for tumbling (**a,b**) and spinning (**c,d**), indicated by the subscript *t* and *s* respectively, and reported as a function of the dimensionless curvature $K^{*}$ and the angular displacement $\theta$. The angular displacement is computed using the rotational matrix (**a,c**) and quaternions (**b,d**) for a fiber resolution of 100 voxels

The random error, $\sigma /\sqrt{N}$, is significantly influenced by the fiber’s parameters, particularly resolution and curvature. Resolution exerts a stronger influence on $\sigma$ than curvature. In terms of the rotation estimation method, lower values of $\sigma /\sqrt{N}$ are observed with the Rotational Matrix (RM) method. However, as resolution improves, the difference between the RM and quaternion methods becomes less pronounced. Moreover, the applied tumbling rotation shows minimal dependence on the random error, as indicated only by the curves with low resolution and small angular displacement in Fig. 7b. In contrast, a power-law relationship of $\sigma /\sqrt{N} \sim \theta _s^{0.5}$ is observed for spinning rotation at small angles ($\theta _s < 3^\circ$), which remains consistent even for fibers with higher resolutions.

From these considerations, it is evident that the absolute systematic error of $\theta _m$ can be reduced by reducing the angular displacements until small angles are achieved. In the context of measuring a specific flow, this is analogous to decrease the time interval between camera exposures, within certain limits. In addition, the systematic error exceeds the random error only at larger angles ($\theta > 10^\circ$). The fiber resolution appears to be the most significant parameter influencing the random error. A more detailed analysis of the effect of curvature is presented in Fig. 8, which displays the two-dimensional contour of the total error, described as follows:

12 $$\begin{aligned} \delta =\sqrt{\mu ^2+\left( \frac{\sigma }{\sqrt{N}}\right) ^2}. \end{aligned}$$

For both tumbling and spinning rotations, the relative total error ($\delta _t/\theta$ for tumbling and $\delta _s/\theta$ for spinning) exhibits a global minimum at a specific angular displacement $\theta$, which can be defined for each fiber curvature $K^*$. For angular displacements exceeding this minimum, the contribution of the systematic error, as discussed earlier, becomes dominant. In this region of the map, the isolines of $\delta /\theta$ appear vertical, indicating that the error is completely independent of $K^*$. For tumbling rotation, the minimum value slightly decreases with increasing curvature (approximately from 0.2% to 0.05% as $K^*$ decreases from 0.6 to 0.05). This reduction is attributed to the fact that for more curved fibers, the eigenvector $\lambda _1$ of the principal axis $\textbf{e}_1$ decreases and becomes more susceptible to noise in the orientation estimation. Since tumbling rotation is primarily dependent on changes in the orientation of $\textbf{e}_1$, this noise has a greater impact. In addition, it is demonstrated that for small angular displacements $\theta$, the rotational matrix (Fig. 8a) yields lower relative error values in respect with quaternion method (Fig. 8b). For example, for a fiber with $K^* = 0.3$ rotating by $\theta = 0.5^\circ$, the relative total error $\delta _t/\theta$ is 1% when using the rotational matrix, whereas it is 1.6% when using the quaternion method. This slight difference is attributed to the additional forward and backward transformation steps required by the quaternion method. For spinning rotation, which is more strongly influenced the secondary eigenvalues, ($\lambda _2$ and $\lambda _3$), the global minimum value of $\theta$ occurs at higher curvature values, where those eigenvalues are larger and less influenced by orientation noise. In addition, for very low curvature values ($K^* < 0.05$), the relative errors appear independent of $\theta$. Finally, similar to the tumbling case, the rotational matrix method slightly outperforms the quaternion method in retrieving more accurate values of rotation rates for small spinning angles.

In conclusion, it was observed that the differences between the quaternion and rotational matrix methods for calculating rotational rates are minimal, particularly when considering the need to maintain total error at a minimum. To achieve this, angular displacements larger than 10$^\circ$ between two consecutive steps should be avoided, and the selection of the time interval should account for this consideration. Additionally, in assessing measurement uncertainty using synthetic fibers, reconstruction noise was not included in the analysis. As it will be further discussed in the next section, this noise can play a significant role in real data.

## Proposed procedure

**Fig. 9:**
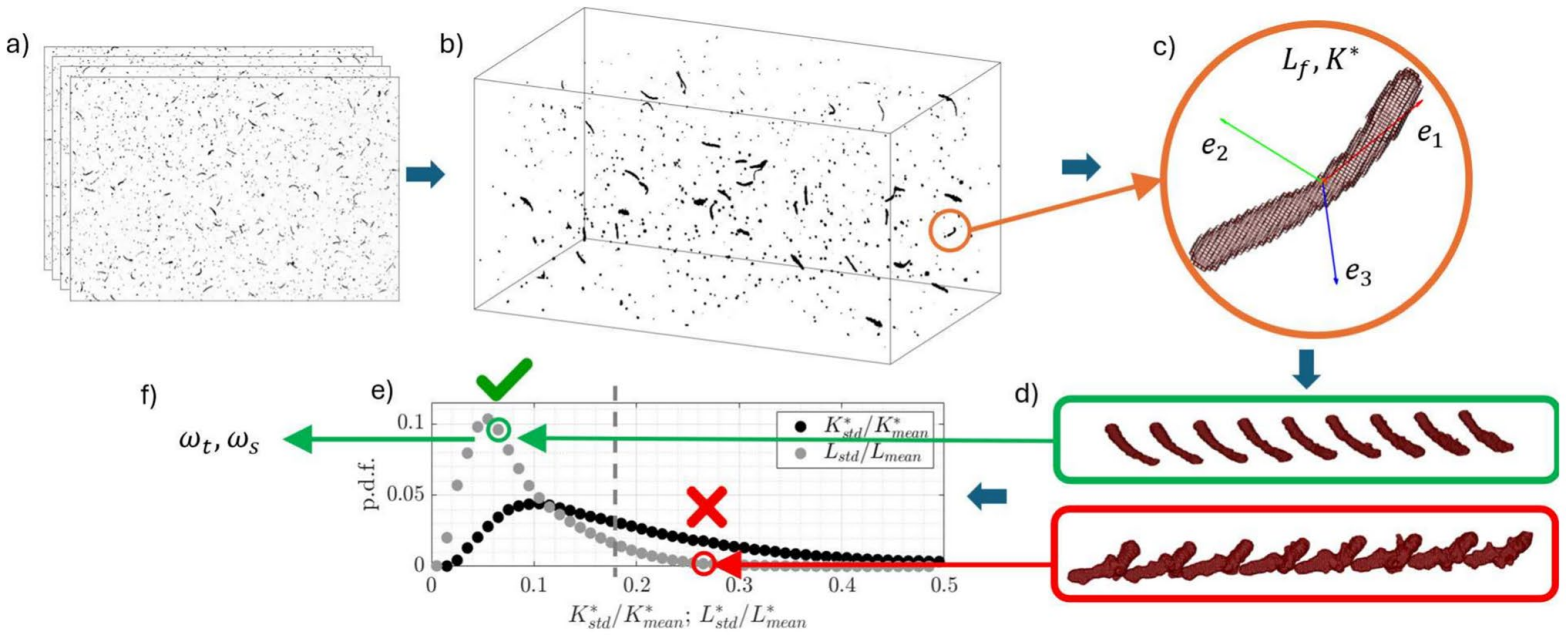
Overview of the methodology used for fiber reconstruction, including image acquisition (**a**), 3D reconstruction (**b**), extraction of relevant geometric parameters (**c**), tracking (**d**), and application of a geometric filter (**e**) before computing the rotational rates (**f**)

The procedure for reconstructing, characterizing, and tracking the fibers, as well as computing their rotation, is presented here. This method, sketched in Fig. 9, integrates both commercial and in-house codes, as detailed in Alipour et al. (2021). Modifications have been applied to this framework to minimize sources of measurement error, which will be discussed in this section. Below, the experimental setup used to retrieve the actual trajectories of fibers in a turbulent water channel is described. Afterward, the algorithm is summarized.

### Experimental and imaging setups

The measurements are conducted in the TU Wien Turbulent Water Channel at $Re_{\tau } = 720$. The system consists of a duct that is 10 m long, 800 mm wide and 80 mm high (aspect ratio 10), where water is used a fluid. The main components of the system including the channel geometry, control system, and imaging setup are fully described in Giurgiu et al. (2023), while the flow parameters and fiber characteristics are defined in Giurgiu et al. (2024b). In the fully developed turbulent region of the channel flow, the viscous length and timescales are $\delta _\nu =55$ $\mu$m and $\tau _\nu =2.7$ ms. The Kolmogorov length and time scales vary between the wall and the channel center in the range $\eta =83-286~\mu$m and $\tau _\eta = 5.7-75$ ms, respectively. The values for the Kolmogorov length and time scales have been obtained by interpolating between values obtained through direct numerical simulations of turbulent channel flows at $Re_\tau =590$ (Moser et al. 1999) and $Re_\tau =950$ (Del Alamo et al. 2004). The fibers have a density of $\rho _f = 1.15$ g/cm$^{-3}$, a length of $L_f = 1.2$ mm, and a diameter of $D_f = 10~\mu$m. Relative to the local Kolmogorov length scale, their length $L_f$ ranges between $4.2 \eta$ and $11.4 \eta$, spanning from the channel center to 2 mm above the wall, respectively. The fibers are dispersed in water, forming a dilute suspension with a volume fraction of $10^{-9}$, resulting in negligible interparticle interactions and minimal influence on the flow.

The imaging system consists of six high-speed cameras (Phantom VEO 340 L) and the fibers are laser-illuminated (Nd:YAG Litron LD25-527) for better visualization (further details are available in Giurgiu et al. 2023, 2024b). We adopted a laser, but alternative solutions can be implemented to ensure good contrast between the fibers and the background.

### 3D reconstruction of fibers

The fibers reconstruction process consists of three main steps (namely MART reconstruction, phase discrimination, and determination of the geometrical parameters), which we summarize in the following.

For each time-step one image per camera is acquired and pre-processed (spatial and time filtering) using DaVis 10.2.1 (LaVision GmbH). The three-dimensional light intensity distribution is reconstructed from the images using a Multiplicative Algebraic Reconstruction Technique (MART) (Elsinga et al. 2006). Each voxel within the reconstructed three-dimensional object corresponds to one of the following: a fiber, a tracer, an optical disturbance, or a numerical artifact introduced by the MART algorithm. To accurately identify the voxels associated with fibers, a shape-based discrimination method is applied. Specifically, objects are classified as fibers if their length, measured in voxels, exceeds a defined threshold. This threshold is determined by the actual fiber dimensions and the magnification level of the imaging system.

To determine the fiber’s morphology, the *regionprops3* function in MATLAB 2023 is used, which provides key geometrical parameters of the 3D reconstructed fiber, such as length ($L_f$), volume, and center of mass, all in voxel units. Before applying this function, it is essential to binarize the 3D object representing the fiber. The MART algorithm produces a voxel distribution with an intensity gradient, where the fiber is characterized by higher-intensity voxels along its core and lower-intensity voxels at its interface. Therefore, an intensity threshold is required to set voxel values inside the fiber to 1 and surrounding values to 0. The threshold value is selected by visualizing the resulting binarized reconstructed fibers and verifying if they maintain their original shape. It was observed that excessively high threshold values resulted in cut and deformed fibers, while values that were too low produced noise at the interface. To determine the orientation of the fiber with respect to the laboratory reference frame ($\textbf{R}$) the principal axis method is used, as described in Eq. (6). The principal axes are obtained through eigenvalue decomposition (Eq. (2)).

### Lagrangian tracking

Each fiber is tracked using a nearest-neighbor scheme, which involves locating its center of mass within a specified radius across two consecutive snapshots (Maas et al. 1993). This radius, defined in voxel units, is chosen based on the experimental setup and flow characteristics. The temporal evolution of the fiber’s position (centroid) is then used to calculate its translation velocity. This relatively simple yet robust method is feasible when the fiber concentration is very low (approximately 100 fibers within the measurement volume) and when the mean displacement is significantly smaller than the mean distance between fibers.

During the post-processing of Lagrangian tracking velocimetry data, experimental noise can be minimized by using temporal filters. Generally, positional errors can be reduced by nearly half when applying a well-calibrated filter (Schröder and Schanz 2023). Several methods can be applied for this purpose, including second-order polynomial filters (Berk 2024), filters with strength adjusted via kernel length and dynamically adapted to the particle’s state (Janke and Michaelis 2021), B-spline filters calibrated to local noise conditions (Gesemann 2021), or those based on probability models (Kearney et al. 2024).

**Fig. 10:**
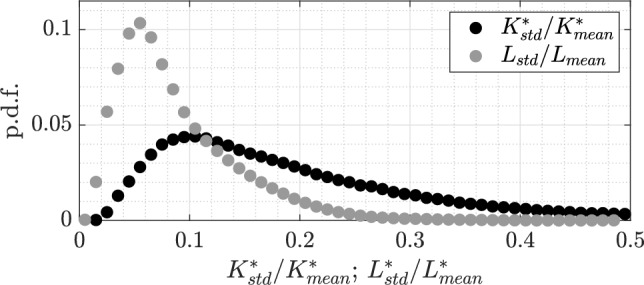
Probability density function of the standard deviations of $L_f$ and $K^*$, computed along 16,000 trajectories normalized by the mean

**Fig. 11:**
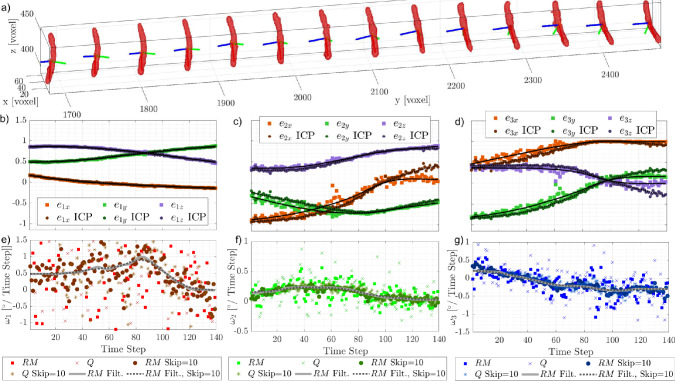
Example of a small fiber ($L \approx 90$ voxels). **a** Segment of the reconstructed trajectory, every 8th fiber is shown. **b**–**d** Orientation matrix components: squares represent values from the reconstructed fiber (solid lines: filtered orientations), circles denote ICP-derived values (dotted lines: filtered orientations). **e**–**g** Rotational rates around the principal axes from the Rotational Matrix (RM) and quaternion (Q) methods. Temporal skipping and filtering effects are shown: solid gray for RM filtering, dotted for RM with a skip factor of 10

### Geometrical filtering

The error associated with the reconstruction noise can be readily detected once the trajectories are determined. Since the fibers can be considered rigid in these flow conditions (Alipour et al. 2022), their geometry remains unchanged over time. Therefore, quantities such as $L_f$ and $K^*$ should not vary along each trajectory. It follows that, for example, large values of the standard deviation of the curvature, $K^*_{std}$, or of the length, $L_{std}$, calculated along a trajectory indicates that fiber reconstruction is failing to accurately resolve the fiber shape. Consequently, estimations of the principal axes and orientation are likely already influenced by noise, as described in Sect. 2.

Figure 10 presents the probability density functions of $K^*_{std}$ and $L_{std}$, each normalized by the corresponding mean values computed separately for each trajectory. The dataset considered contains approximately 16,000 trajectories. Among these, there are trajectories in which the geometry fluctuates by more than 40%, and curvature and length more than and 20%. Therefore, it is advisable to discard trajectories (or a portion of them) where the standard deviation of any geometrical quantity exceeds a chosen threshold relative to its mean value. In the present case, it was chosen 20% for $K^*$ and 10% $L_f$.

**Fig. 12:**
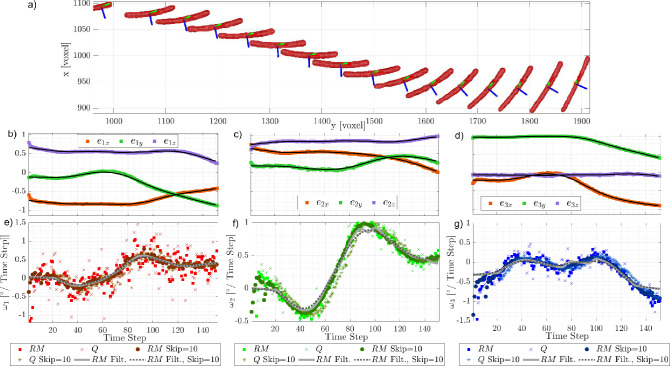
Example of a large fiber ($L\approx 135$ voxels). **a** Segment of the reconstructed trajectory, every 8th fiber is shown. **b**–**d** Orientation matrix components: squares represent values from the reconstructed fiber (solid lines: filtered orientations). **e**–**g** Rotational rates around the principal axes from the Rotational Matrix (RM) and quaternion (Q) methods. Temporal skipping and filtering effects are shown: solid gray for RM filtering, dotted for RM with a skip factor of 10

### Gyro tracking

After determining the trajectories and filtering out those affected by excessive 3D reconstruction noise using the previously described methods, the fiber’s orientation and rotational motion can be measured-a process known as *gyro tracking* (You and Neumann 2001).

Figure 11a shows a portion of the trajectory of a real fiber ($L_f \approx 90$ voxels, $K^* \approx 0.27$), where the three-dimensional distribution of the voxels forming the fiber is visible at each time step. The orientation of the fiber is computed using Eq. (3), and the nine components of the rotation matrix are plotted over time in Fig. 11b–d. These components exhibit fluctuations, which primarily stem from geometric imperfections in the fiber reconstruction.

As expected, larger fluctuations are observed in the components corresponding to the secondary principal axes, $\textbf{e}_2$ and $\textbf{e}_3$, due to the slender shape of the fiber. In this case, the eigenvalues $\lambda _2$ and $\lambda _3$ are much smaller than $\lambda _1$, making their associated orientation vectors more susceptible to noise. Notably, changes in the orientation of these secondary axes directly contribute to the spinning rate, $\omega _1$. Consequently, as shown in Fig. 11e, the spinning rate exhibits the highest level of scatter among the measured rotational rates.

This behavior is analogous to translational velocity measurements in particle tracking velocimetry (PTV), where noise in the particle position affects displacement estimation and, consequently, velocity measurements. To mitigate these effects, temporal filtering is commonly applied. Similarly, for rotational rates calculations, a robust locally weighted scatterplot smoothing (’rlowess’) filter (Cleveland 1979) is applied to the all nine components of the rotation matrix $\textbf{R}$ before using Eqs. (5) and (6). This method, previously employed by Shaik et al. (2020), Shaik and van Hout (2023), Giurgiu et al. (2024b, 2024a), is particularly effective in handling outliers compared to traditional smoothing techniques, such as Gaussian or second-order filters (Bowman and Azzalini 1997). First, the components of $\textbf{e}_1$ and $\textbf{e}_2$ are filtered using a kernel of 40 time steps. Since this filtering process disrupts the orthogonality of the vectors, the filtered $\textbf{e}_3$ is reconstructed as the cross-product of the filtered $\textbf{e}_1$ and $\textbf{e}_2$. Finally, $\textbf{e}_2$ is recomputed as the cross-product of $\textbf{e}_3$ and $\textbf{e}_1$, ensuring that the resulting vectors form an orthonormal system. The effect of this filtering can be observed in Fig. 11b, where the black lines represent the smoothed (rlowess’-filtered) trajectory. The advantage of the filtering process becomes significantly more evident in the final plot of the rotational rates, as both the rotation matrix and quaternion approaches are highly sensitive to noise in the $\textbf{R}$ components.

Figure 11e–g also shows that the rotational rate components exhibit only minor differences when comparing the rotation matrix and quaternion approaches. However, a higher degree of scattering is observed in the values obtained using the quaternion approach compared to the rotation matrix. As discussed in the previous section, for small angular displacements (one degree or less), the quaternion approach is less precise in retrieving rotational rates (Figs. 7 and 8) for both synthetic and real fibers. Notably, the systematic behavior observed for small angles in the synthetic data is also present in the experimental results.

As shown in Sec. 3, another approach to reduce errors consists of increasing the angular displacement, which can be achieved by lowering the acquisition frequency or applying a time skip *a posteriori*. In Fig. 11e–g, a time skip of 10-corresponding to less than a Kolmogorov length scale at the fiber’s location-produces results (circle markers) that closely match those obtained with the time filter (solid gray line). Notably, the spinning component, $\omega _1$, exhibits consistently higher scattering than the other two components. As expected, increasing the angular displacement (i.e., applying a time skip) brings the $\omega$ values closer to those obtained with the time filter, confirming the validity of both post-processing approaches in the absence of artifacts. Of course, both techniques have the drawback of reducing sensitivity to fluctuations smaller than the kernel time scale of the filter and the time skip.

If a larger fiber is used, with its length increased by a factor of 1.5 and its diameter by a factor of 3, and it is reconstructed using the same imaging settings (i.e., the same magnification factor), the influence of interfacial noise on the rotation matrix $\textbf{R}$ is significantly reduced, as shown in Fig. 12b–d. Consequently, the rotational rates also exhibit lower sensitivity to noise, as illustrated in panels **e** to **g**. Similarly, as observed in Sec. 3, the errors retrieved for larger synthetic fibers (Fig. 7) are lower than those obtained for smaller fibers. Although the scattering of the resulting rotational rates is reduced, the same trends observed for small fibers persist. Specifically, small differences remain between the rotation matrix and quaternion approaches, the time filter reduces fluctuations, and increasing the angular displacement further decreases scattering. However, the scattering of $\omega _1$ remains higher than that of the other components. As previously discussed, this is due to differences in eigenvalues and is independent of image resolution (voxel size). Nevertheless, increasing the fiber size appears to be the most effective way to improve the experimental accuracy of these measurements.

**Fig. 13:**
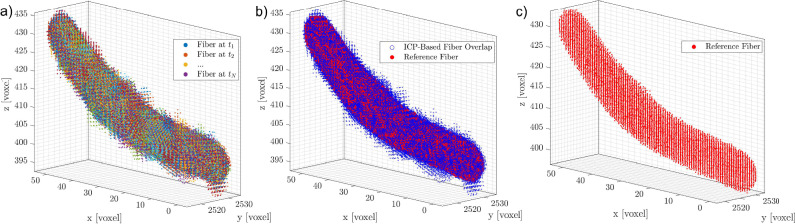
**a** Superimposition of all point clouds corresponding to each time instant for the small fiber (90 voxels) analyzed in Fig. 1. **b** Comparison between the reference fiber (red) and the superimposed point clouds of all fiber instances (blue). **c** Shape of the reference fiber (ICP) used to compute the rotational rates in Fig. 11

**Fig. 14:**
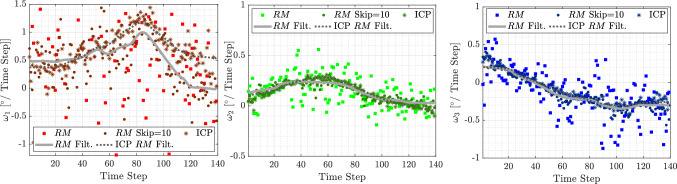
Effect of the ICP algorithm on the rotational rates $w_i$ and comparison with the temporal skip

However, increasing magnification is not always feasible, as it significantly reduces the measurement volume, limiting the trajectory length and Lagrangian statistics. Similarly, using larger fibers is not always an option. An alternative approach to mitigate the interfacial noise affecting is to employ an advanced registration algorithm. In this context, the iterative closest point (ICP) algorithm provides a promising solution. It could effectively reduce the random error in the orientation matrix (Fig. 11b–d dark circles). In particular, this algorithm enables the generation of a *reference fiber* by superimposing all 3D instantaneous reconstructions of a fiber along its trajectory (Fig. 13a). This was downsampled using a voxel-based approach to reduce noise and computational complexity while preserving the overall shape: the 3D points cloud of the reference fiber was centered by computing the mean position of all points in a unit volume of 1 voxel (see Fig. 13c). This process is able to reduce the interface noise of the fiber as shown in Fig. 13b. The same algorithm is then used to align the reference fiber with each instantaneous reconstructed fiber. The orientation is determined by minimizing the voxel-based discrepancy between the two, ensuring a more accurate estimation of fiber orientation, as shown in Fig. 11b–d. The resulting rotational rates of the presented techniques are shown in Fig. 14. Although the improvement with the ICP algorithm is not substantial, the scattering in the ICP data with a time skip of 10 is noticeably lower than that observed when applying the same skip to the raw orientation components.

## Conclusions

This study examined the sources of uncertainty in experimental 3D imaging techniques used to measure the full rotational dynamics of curved fibers in turbulent flows. A comparative analysis of two common methods for computing rotational rates-the rotation matrix and quaternion approaches-enabled the identification of key experimental error sources.

A critical factor influencing measurement accuracy is the three-dimensional mass distribution of the reconstructed particles. Reconstruction noise, which is highly sensitive to fiber shape, affects the determination of principal axes and, consequently, the computed rotational rates. To mitigate these uncertainties, time filtering plays a crucial role, potentially even more so than in Lagrangian tracking, as it corrects small positional errors that otherwise propagate into the rotational measurements. Additionally, increasing fiber size, when feasible, significantly reduces interfacial noise, leading to improved measurement accuracy and lower scattering in the computed rotational rates.

A key contribution of this work is the parametric analysis conducted with synthetic fibers, allowing for *a priori* uncertainty quantification and valuable insights into experimental design and data processing. By systematically varying independent parameters such as spatial resolution, curvature, and angular displacement, we demonstrated their impact on measurement errors. The results indicate that systematic errors can be minimized by maintaining an optimal angular displacement-neither too small nor excessively large-while fiber resolution strongly influences random errors. Furthermore, an analysis of dimensionless curvature and angular displacement revealed that systematic errors dominate at larger angular displacements, whereas random errors follow a power-law trend at small spinning angles.

Another key finding is the importance of evaluating fluctuations in the rotation matrix components before computing rotational rates. Increasing the angular displacement, either by reducing acquisition frequency or by applying a time skip, helps to mitigate the effects of noise. Additionally, while the iterative closest point (ICP) algorithm provided only moderate improvements, its combination with a time skip significantly reduced scattering in the computed rotational rates and provides a physically grounded smoothing approach.

These findings enhance the accuracy of fiber rotation measurements in turbulence, addressing limitations in previous studies, particularly in the quantification of spinning rates. The proposed approach-integrating advanced imaging techniques, synthetic data validation, and optimized post-processing strategies-offers a robust framework to mitigate experimental noise and geometric ambiguities in fiber tracking. By refining key parameters such as angular displacement, fiber image size, and filtering strategies, this work contributes to a deeper understanding of anisotropic particle dynamics in turbulence, with implications for industrial applications, environmental monitoring, and micro-plastic pollution research.
